# How to Facilitate Disclosure of Violence while Delivering Perinatal Care: The Experience of Survivors and Healthcare Providers

**DOI:** 10.1007/s10896-022-00371-z

**Published:** 2022-03-22

**Authors:** Ann Pederson, Jila Mirlashari, Janet Lyons, Lori A. Brotto

**Affiliations:** 1 Population Health School of Population and Public Health, University of British Columbia, Vancouver, Canada; 2 Department of Obstetrics and Gynecology, University of British Columbia, Vancouver, Canada; 3 Division of General Gynecology & Obstetrics, University of British Columbia, BC Women’s Hospital, Provincial Health Services Authority (PHSA), Vancouver, Canada

**Keywords:** Perinatal care, Gender-based Violence, Disclosure, Facilitators, Healthcare providers, Women

## Abstract

Gender-based Violence (GBV) during the perinatal period is a serious concern as it is associated with many adverse outcomes for both the mother and the baby. It is well known that violence is under-reported. Thus, official statistics (both police reports and survey data) underestimate the prevalence of violence in general and during the perinatal period specifically. In this study conducted in Canada, we sought to explore the barriers to and facilitators of women disclosing their experiences of GBV within healthcare services to safely facilitate more disclosure in the future and reduce the harms that arise from GBV. We used thematic analysis to analyze in-depth interviews with 16 healthcare providers (nurses, midwives and physicians) and 12 survivors of GBV. The data reflect three main themes: “raising awareness of gender-based violence”, “creating a shift in the healthcare system’s approach toward gender-based violence” and “providing support for survivors and care providers.” Our findings suggest that the healthcare system should increase its investments in raising awareness regarding GBV, training healthcare providers to respond appropriately, and building trust between survivors and healthcare providers. Healthcare providers need to be aware of their role and responsibility regarding identifying GBV as well as how to support survivors who talk about violence. Expanding a relationship-based approach in the care system and providing support for both survivors and health care providers would likely lead to more disclosures.

## Introduction

Gender-based violence (GBV) is violence directed at an individual based on their sex or gender identity. It encompasses a wide range of abuses such as sexual threats, rape, exploitation, humiliation, assault, molestation, domestic violence, incest, torture and depriving women of their right to enjoy their freedom and having control over their life (Blondeel et al., 2018; Sinko et al., 2021). GBV can also affect members of the LBGTQ + population, men, and individuals who identify as gender non-binary (Blondeel et al., 2018; Graaff, 2021) In Canada, GBV disproportionately affects women, girls, Indigenous women, women with disabilities, and women living in rural or remote regions. Also, individuals of diverse gender identities and expression are at greatest risk of violence (Chmielowska & Fuhr, 2017; Government of Canada S. of  W. C., n.d.; Nelson & Lund, 2017; Valentine et al., 2019).

The detrimental effects of violence for mothers and their developing fetuses during the perinatal period are more pronounced than among survivors who experience violence outside the perinatal time (Taillieu et al., 2016). The perinatal period in this study is defined as the pregnancy period up to a year after giving birth (Garcia & Yim, 2017). In fact, violence during this time is a serious concern; it is associated with adverse outcomes such as premature birth, miscarriage, low birth weight, admission to a neonatal intensive care unit, sexually transmitted infections (Paterno & Draughon, 2016), depression, substance use and financial difficulties (Taillieu & Brownridge, 2010).

Unfortunately, GBV is under-reported; in 2014, fewer than 19% of those who had been abused by their partner reported the incident to police. Consequently, most cases of abuse and violence are not captured in official statistics (Family Violence in Canada: A Statistical Profile, 2014, 2014). A recent report suggests that more than one-third of survivors wait for more than two years before disclosing the abuse (Boethius & Åkerström, 2020). The prevalence of violence during pregnancy in developing countries is estimated as 27.7% and in developed countries as 13.3% (Stewart et al., 2017). Indeed, the highest prevalence of violence is reported among women of reproductive age, with the highest rates occurring among individuals 18 to 34 years old. Clearly, women in their perinatal period are not immune from GBV. Therefore paying special attention to GBV within the reproductive health setting is essential. (Bair-Merritt et al., 2014; Taillieu et al., 2016) These data demonstrate the need to focus attention on GBV during the perinatal period (Hahn et al., 2018).

To assess the effectiveness of screening for violence within health care settings on identification, referral, and re-exposure of violence and evaluation of women’s health outcomes, Doherty et al. did a review. They included 13 trials that recruited 14,959 women from diverse healthcare settings. The settings were predominantly located in high-income countries and urban areas. They explained that women during their perinatal care might be more likely to disclose GBV when screened for experiences of violence. However, there is no evidence showing that screening by itself impacts other outcomes such as referral, re-exposure to violence and health measures when not accompanied by interventions that can support the victim. Thus, while identification of GBV increases by screening, there is insufficient evidence to justify screening in healthcare settings (O’Doherty et al., 2015). Although most studies emphasize the importance of disclosure, there is still no consensus among all diverse schools of thought regarding screening. British Columbia (BC) Women’s Hospital & Health Centre does not currently conduct screening for violence because staff and leaders lack training. Also, they believe in BC, there is limited infrastructure for GBV services. At present, the hospital’s policy is to inquire regarding violence when there are signs that it may be present rather than to offer universal screening. This case-finding approach is supported by trauma- or violence-informed approaches to perinatal care (Rossiter, 2011). In agreement with this approach, WHO mentioned that there is insufficient evidence supporting the idea that screening reduces GBV or improves the quality of life or health outcomes (MacMillan et al., 2009).WHO also stipulates the necessity of certain conditions before application of GBV screening, such as access to trained health care providers who can speak to women privately and are able to provide post-screening interventions. Therefore, screening application is difficult in some clinical settings with low resources and lack of training (Bacchus et al., 2010; MacMillan et al., 2009; O’Reilly & Peters, 2018).

Although the health sector is one of the primary resources for women who have survived abuse, the perinatal care providers’ role in GBV intervention programs is currently limited (Purwaningtyas et al., 2019). While care providers agree that violence against women is a healthcare issue, it is nevertheless often overlooked in clinical settings (Ramsay et al., 2012; Usta & Taleb, 2014).

The literature suggests several factors contribute to healthcare providers’ limited assessment of GBV in their practice, including providers’ personal discomfort with the topic; perceived inadequate resources to address GBV; lack of time; and lack of training (Hegarty et al., 2020). Many clinicians feel poorly prepared to ask relevant and sensitive questions about GBV; many reports not having access to a private place to raise the topic (Paterno & Draughon, 2016). Other documented barriers include the perception that assessing violence is not the clinician’s role; fear of offending women; and uncertainty regarding how to respond to a disclosure of violence (Paterno & Draughon, 2016; Portnoy et al., 2020).

Women themselves have identified various reasons for not disclosing experiences of violence during the perinatal period, including self-blame; shame; fear of the consequences; lack of knowledge of services (Shaheen et al., 2020); concerns that child protection officials will become involved; fear of not being believed; and fear that disclosure might escalate or exacerbate the violence (Curry et al., 2011). There is some literature on the effective facilitators of GBV disclosure. For example, many of the barriers can be addressed through proper training and the development of a systematic inquiry protocol (Paterno & Draughon, 2016). A qualitative study was conducted in UK in London’s mental health centers to explore the facilitators and barriers to domestic violence disclosure from a service user and professional perspective. Both healthcare providers and survivors reported that supportive and trusting relationships between clients and care professionals would facilitate disclosure (Rose et al., 2011). The extent to which survivors perceived they would be deemed credible and would receive tangible emotional support from care providers have also been identified as facilitators of disclosure (Curry et al., 2011). Therefore, communicating with compassion, providing information and asking questions in a private, safe and supportive atmosphere, and explaining why questions have been asked are reported to help women feel more comfortable disclosing (Chang et al., 2005). In these supportive circumstances, survivors might report experiencing less stigma when sharing their stories with healthcare providers (Chang et al., 2005; Murray et al., 2016).

Previous research on GBV and healthcare has not typically focused on the perinatal period. Receiving first-hand information from survivors and care providers regarding facilitators of the disclosure may help decision-makers design interventions and programs that are more compatible with the existing context. This study aimed to learn from survivors’ and healthcare providers’ experiences about facilitators of disclosure during perinatal healthcare in BC.

## Methods

### Participants

In qualitative research, data saturation will determine the number of interviews. In this study, we interviewed 28 participants, 16 healthcare providers (nurses, midwives and physicians) and 12 survivors.

### Setting

Healthcare providers were recruited from maternity services and NICUs in different parts of British Columbia. The survivors were from several various metropolitan centers in British Columbia.

### Inclusion and Exclusion Criteria for the Survivors

being between the ages of 18–49, having been pregnant during the last five years, having a self-reported history of GBV in the perinatal phase, and communicating and speaking conversational English. In a short pre-interview session, survivors were asked about the experience of current drug dependency and severe mental health problems. Since the experience of current drug dependency and major mental health problems could influence the experience of GBV, self-reports of current alcohol or drug dependency and major mental health problems (such as major depression and severe bipolar disorder) have been considered as exclusion criteria.

However, women who had an experience of drug dependency in the past and those who were involved in the recreational use of drugs were not excluded. In order to consider maximum variation, survivors with diverse identities (including those identifying as Indigenous and immigrants) were recruited.

### Inclusion Criterion for the Healthcare Providers

Having the experience of providing perinatal healthcare to women with a history of GBV. Healthcare providers were recruited from different parts of British Columbia.

### Procedures

For participant recruitment, flyers and advertisements in settings such as hospitals and public places like community centers, shelters, shopping malls, gyms, family services, and courts were distributed. Social media advertisements (i.e., Instagram and Facebook) and snowball sampling were used to recruit additional participants. An office telephone number and email were provided in the advertisement for potential participants to contact the researcher.

Before the interviews, demographic characteristics, inclusion and exclusion criteria were assessed by a short telephone conversation, and comprehensive information about the study was provided. Participation in the study was voluntary, and the same recruitment method was used for both groups. Before the interview, consent was received from the participants, and they were assured that their personal information, transcripts, and recorded files would be anonymized.

Interview questions were designed by one of the researchers (JM) and finalized after discussion and exchange with the team members. Semi-structured interviews were conducted by JM, who has extended experience in qualitative research and interviewing. Out of 28 interviews, ten were carried out in person (in a cafeteria or healthcare facility), and after COVID -19 pandemic, interviews were conducted via phone. Women were asked about their experiences regarding violence and the responses they received from the health care system. Both groups were asked about underlying reasons that made some of the survivors break the silence and talk about their experience of violence with health care providers. We asked about their suggestions for improving the existing context of identifying GBV within the health care system. Furthermore, care providers talked about their approach toward the assessment of GBV. (Tables 1 and 2 show examples of semi-structured questions in interviews with care providers and survivors).

**Table 1:** Examples of semi-structured questions in interviews with survivors

1. How do you define GBV?
2. Who was the first person that you decided to talk about the experience of GBV with?
3. Have you ever had the experience of asking for help from a health care provider? If yes, please explain what made you disclose it to a healthcare provider?
4. Over the course of pregnancy and after that, did anyone in the health care system ask you about your history of abuse or violence?
5. What are the facilitators to disclosing violence based on your experience?

**Table 2:** Examples of semi-structured questions in interviews with healthcare providers

1. How do you define GBV?
2. Have you ever come in contact with cases of GBV among your clients? Please explain how did you notice that and what was your response?
3. Over the course of pregnancy and after that, do you usually assess your clients for a history of violence/abuse?
4. What is the role of healthcare providers regarding GBV?
5. Based on your experience, how is it possible to increase the care provider’s level of involvement in the identification of GBV?
6.How can healthcare providers encourage disclosure?

### Ethical Consideration

The UBC C&W Research Ethics Board approved this study (Approval number: H19-02,409). The researchers ensured that the appropriate procedures were followed regarding informed consent, anonymity, autonomy, and maintaining confidentiality.

### Data Analysis

A qualitative approach was used to explore participants’ thoughts, experiences, and recommendations regarding facilitators of disclosure. Twenty-eight interviews were conducted. Interviews lasted between 30–60 min. Data collection and analysis occurred simultaneously; each interview audio file was transcribed verbatim and analyzed, and then the next interview was conducted. After each interview, the researcher wrote reflective notes and memos. This iterative approach is recommended to enhance the value and quality of the findings as subsequent interviews are informed by previously collected data (Suter, 2012). Simultaneous analysis helps the researcher to be more confident about obtaining rich, deep and related data so that the researcher learns from each interview and its analysis to have a better interview with the next participant and would be able to cover the gaps (Suter, 2012).

A thematic, inductive approach was used to analyze the data (Nowell et al., 2017) using NVIVO. All interviews were conducted by one of the team members. Two of the researchers who were expert qualitative researchers did the analysis separately and then discussed their themes with the rest of the research team. The entire text was read several times to identify meanings or possible patterns. We conducted line-by-line coding to stay close to the data and preserve the action and language represented in the text. After that, initial codes were identified, and the data were organized into meaningful groups and sorted into potential themes and subthemes. Moreover, themes and subthemes were reviewed in terms of internal and external consistency. Based on the suggestions of other team members, some themes were modified, and a short and concise name was chosen so that the readers would understand each theme. The analysis suggested that both survivors and healthcare providers shared a number of similar perspectives, and we, therefore, present the findings from both groups together.

To support the trustworthiness of the findings, Lincoln and Guba’s proposed criteria, including credibility, confirmability, dependability, and transformability, were considered (Nowell et al., 2017). Strategies used to support trustworthiness included repeated readings of the transcripts and prolonged engagement with the data. Moreover, emerged themes and subthemes were reviewed with team members. To prevent insider bias, the researcher practiced reflexivity and documented personal reflections in a project diary. Also, transferability increased by a complete description of participants and process. In our report, we followed the Consolidated Criteria for Reporting Qualitative Health Research (COREQ) so that readers can assess the credibility, dependability, transferability, and confirmability of the study findings (Tong et al., 2014).

## Results

### Participant Characteristics

Twelve interviews were conducted with survivors who had experienced pregnancy within the last five years; the mean age of these women was 35 years old. In order to consider maximum variety, we interviewed survivors with different backgrounds, ethnicity and sociodemographic status. Sixteen interviews were also conducted with healthcare professionals who had provided care for women with a history of GBV (five nurses, five midwives, and six physicians including family physician, OBGYN and a neonatologist). The mean age of care providers was 40 years old. Demographic characteristics of the participants are presented in Tables 3 and 4.

**Table 3:** Demographic characteristics of health care providers

Profession	Age range	Years of experience as a HCP
Physicians	35–54	8–28
Nurses	29–50	8–20
Midwives	34–59	2–20

**Table 4:** Demographic characteristics of survivors

	Age	Ethnicity	Education Level
1	21	Latina	Undergraduate student
2	35	European	Master’s degree
3	41	Indigenous	Elementary
4	40	Indigenous	High school
5	27	South Asian	Undergraduate student
6	38	Euro-Canadian	Master’s degree
7	37	Latina	Bachelor degree
8	39	Euro-Canadian	College
9	40	Euro- Canadian	College
10	35	East Asian	Bachelor degree
11	27	Euro- Canadian	Diploma
12	42	Euro- Canadian	University (not finished)

### Thematic Findings

**Fig. 1:**
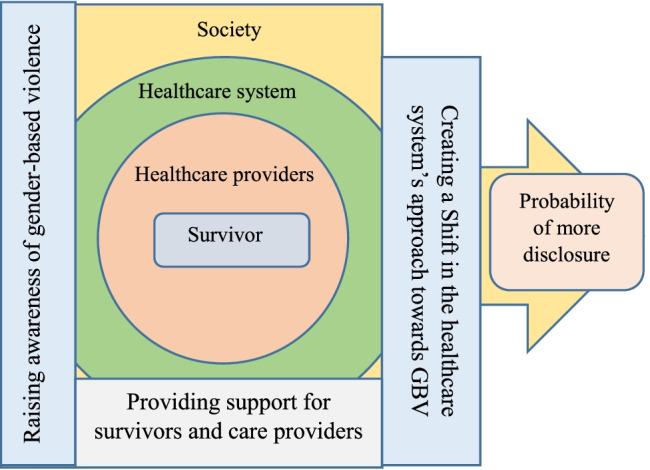
The diagram of facilitators of disclosure

The data can be clustered into three main themes and 13 subthemes that all refer to how to improve the response and system. (Table 5 shows the main themes and subthemes) (Fig. 1 - the diagram of the main findings).

**Table 5:** Overview of 3 major themes and 13 sub-themes

**Raising awareness of gender-based violence**	-De-stigmatization of GBV disclosure in the society - Validate survivors’ experience -Reform gender inequality in society - Create a positive attitude towards the supportive role of the healthcare system by raising awareness
**Creating a Shift in the healthcare system’s approach towards GBV**	-Adopt a relationship-based approach to healthcare -Ensure continuity of care during perinatal period -Establish a safe and secure atmosphere for disclosure - Establish a multicultural approach - Eliminating time constraints and work overload in the healthcare system -Clarify the role of healthcare providers with regard to GBV
**Provide support for survivors and care providers**	-Health care providers training - Support healthcare providers by paying the fee for the service - Providing insurance coverage for the counselling services

### Main Theme #1: Raising Awareness of Gender-Based Violence

The need to raise awareness in the community was one of the common topics mentioned by both groups of participants. Study participants suggested that greater awareness of GBV could change both individual survivors’ as well as broader societal views toward disclosure, thereby encouraging survivors to seek help and support.

“I think what would play a big role is more awareness, like people talking more about it, like how we see ads in the film, in the movies or like when you are watching TV in the evening. I think just having a small one-minute ad, what GBV is? Have you ever suffered, you know, violence or just the community needs to get more awareness? I think that is, you know, media, newspaper, Internet.” (35 yr old Physician).

“I think certainly education is important. We can inform them and tell them these are examples of violence, and this is what it sometimes can look like, or these things could happen to you and are you interested in getting more resources or learning more about this? And there are some places you can go to learn more about this.” (48yr old Physician)

*“I talked about it with everybody with friends and neighbours and family, and nobody identified it as abuse. Everybody just said, oh, well, he is an idiot, or oh, he was a jerk, or it probably was your fault. I would have been out of the relationship for a while that I found resources online. Actually, that made me realize that this is abuse. And then I got a counsellor to help me with it. I did not know until way after it happened; I did not even recognize it as abuse.”* (41yr old Survivor).

#### Reducing the Stigma of GBV Disclosure

Participants noted that the stigma of disclosure makes women stay silent. They mentioned that there should be more effort to de-stigmatize GBV disclosure in society

*“We need to talk about it. I guess in our society it is easier to talk about suicide and depression than GBV.”* (34yr old Midwife).

*“So I would definitely say society does not want to hear about it just like most things with the status quo. And there are fewer people in the middle of being open to it.”* (36yr old Midwife)

#### Validate Survivors’ Experiences

Participants were concerned about not being believed, and health care providers confirmed this concern. Perhaps a lack of awareness about GBV in the community and the healthcare system contributes to doubt toward survivors. Participants spoke about the importance of empathy and receiving respectful, non-judgmental responses from health care providers and society.

*“Being believed is another issue, the fear that someone will not believe you. That is really all I can think of, in my particular case.”*(42yr old Survivor)

*“When she went into labour, it was a really hard situation. She always was screaming a lot every time anybody tried to touch her. The hardest thing was that while this was happening, the other health care providers were not very supportive. They did not understand what was going on, and they were making fun of her. Essentially. The anesthesiologist said, ‘well, you just need to let us know how dilated you are to get the epidural.’ Right? So withholding care from her.”* (36yr old Midwife)

#### Reform Gender Inequality in Society

Referring to a society in which men still have the upper hand, participants explained that, in their experience, this fact could be a barrier to disclosure. They believed that gender inequality in society needed to be reformed.

*“Traditionally, it is predominantly a male-influenced society and women are often silenced over hundreds and hundreds of years. There is still some of that present in today is society, just because that culture has been around for so long. So, I think that we are more supportive than we have been in the past, but we are still not fully viewed as equally or want to be heard as equally.”* (29yr old Nurse)

*“They just did not recognize it as something serious. They just kind of thought, well, men behave like men, you know. It is just this cultural and social thing about ‘oh you’re probably going to be mistreated, and he is not hitting you, so it is okay.”* (41yr old Survivor)

*“I think it is a very male dominant society. Very, you know, white-dominated. So I think it is more than just gender. I think that there’s a lot of sort of domination and inequality…but I think being a minority also, you know, is kind of like a double hitter.”* (36yr old Midwife)

#### Create a Trusting Relationship Between Care Provider and Survivor by Raising Awareness

Based on the participants’ experiences, many survivors were unsure about receiving support from the health care system because they thought the healthcare system’s focus was on physical problems. At the same time, they were concerned that the care providers would report the violence to the police. Fear of losing their children and financial hurdles also prevented survivors from disclosing the violence. Therefore, creating a positive dynamic and trusting relationship between the health system and survivors through raising awareness plays a crucial role in disclosure.

*“They know that health care providers have the duty to report, and it leads to very serious legal ramifications for their families. So they do not feel health care providers as safe people who actually have any kind of solutions for them. And so why would you report?”* (35yr old Midwife)

*“I was scared like, “What if they call the ministry? Yeah, that is probably the most common fear, fear of losing children, or losing home, you know, losing the partner*.” (40yr old Survivor).

*“I think women would talk about it if they feel safe to do so. But I understand why a lot of them do not feel safe to do so…you know, they can’t trust the care provider to do anything with the information.”* (27yr old Survivor)

### Main Theme #2: Creating a Shift in the Healthcare System’s Approach Towards GBV

Participants pointed to the need for fundamental cultural changes in the health system’s approach to GBV. They talked about systemic sexism and stressed that this change should be done in multiple layers across the healthcare system.

“It’s a system model of care shift that has to happen as well. It is also about larger education about sexism. Like all the ‘isms’ that kind of allow for GBV to happen within healthcare provider curriculum.” (35 yr old Midwife).

“I think everyone focuses on the medical piece of it, but I think the social support piece and those aspects I think they still do not pay enough attention to, so yeah, I do not think we do a good job to still recognize the importance of it. It’s still under-recognized. I think it speaks to a systemic and cultural sort of shift of us shifting from fixing things to learning about people’s values and their life and what’s going on for them and building relationships, so I think it’s a shift between medicalizing everything to being preventative and focusing on other things”. (45 yr old Physician).

#### Adopt a Relationship-Based Approach in Healthcare System

Some participants criticized the quality of communication between healthcare workers and survivors. They argued that healthcare providers are very task-oriented. Some suggested that efforts are needed to establish a more relationship-based approach to support women being willing to disclose.

“I think the second thing is that the system is not set up for kind of open and kind of relationship building and open based questions. It is set up for task-based oriented things in the fee for services system, so I think we are kind of, it makes it hard to do this type of work”. (37 yr old Physician).

“Of course, the care provider should ask about the history of violence and talk about it with their clients. But it has to be done in a way that is non-judgmental, non-punitive and non-shaming. Without that in place, it is going to do more harm than good. You are gonna completely lose the trust of your clients.” (42 yr old Survivor 12).

#### Ensure Continuity of Care During the Perinatal Period

Some survivors mentioned that visiting different healthcare providers can disrupt the care relationship and trust-building between women and their care providers during perinatal care. The possibility of meeting one specific care provider during the perinatal period can be instrumental in disclosure.

*“So we feel a woman is meeting a new nurse every day or a new doctor every day. It’s really hard to build that trust and talk about these issues*.” (42yr old Physician).

*“It is really a sort of a collection of different doctors and midwives that work together. I just went to the Midwife, and often a client like myself would just see a few different midwives during the course of their prenatal support*.”(38yr old Survivor).

*“I guess another one of my problems is my family doctor is floating all the time. I had one woman, and then she left on mat-leave. Now I see some floater doctors; I just have a rotating group of women doctors, so I do not think many of them are reading back into my charts or anything or trying to put it together; they were just dealing with the appointments as they came. You are only allowed to talk about two things at a time. And they kind of, it feels like they are kind of pushing you out.*”(39yr old Survivor)

#### Establish a Safe and Secure Atmosphere for Disclosure

The participants believed care providers should ask questions related to safety and GBV. However, they stressed that these questions should be asked in a private and safe environment. They also spoke about confidentiality and the quality of the conversation between care providers and survivors. It seems that the healthcare system should take action in creating an atmosphere in which women feel comfortable enough to talk about sensitive topics like GBV.

“The person who is having to disclose their own experience should be disclosing it in a safe environment. So, if I ask someone directly with a cold question, it is unfair to expect them to answer.”(59 yr old Midwife).

“I would never have admitted anything while he was there. I’m still afraid, even calling or sending emails or anything. I feel like he is watching me. I still take precautions.”(37 yr old Survivor).

#### Establish a Multicultural Approach

The participants suggested that health care providers should pay more attention to cultural differences when designing programs for supporting survivors. They discussed the idea that there are different definitions of GBV in different cultures and suggested that care providers need to be more aware of these differences. Having a single approach toward GBV while ignoring the impact of multiculturalism on experiences may limit the disclosure.

* “I think every year, doctors, psychologists, anesthesiologist, midwives, anybody that is going to be providing care to women and trans peoples, should be doing some kind of training, and then also we want to discuss how that affects across race and you know, with cultural safety training led by the people from that community. So if it is Indigenous cultural safety, having it be led by an Indigenous person, if it is Black women, Trans women, having their experience be a part of the training, like the voice for doing the training if that makes sense.”*(36yr old Midwife)

*“There needs to be more education on culture and cultural practices. Within cultures, there are tons of different practices and mannerisms and traditions. So, in westernized, like Canadian culture, something that we see or hear in a different culture might come across as something of a red flag, but it is completely normal for them. Every culture is different. So we have to still provide culturally sensitive care*”. (29yr old Nurse)

#### Clarify the Role of Healthcare Providers with Regard to GBV

Some healthcare providers mentioned that dealing with GBV was not their responsibility, and they used to assume that this was not in their scope of practice. Some survivors also mentioned that addressing GBV is not among the duties of healthcare providers and that they did not know that they could receive support from healthcare providers. Accordingly, healthcare providers’ roles and responsibilities regarding GBV should be discussed during clinical training and continuing education. Clarifying healthcare providers’ roles and responsibilities regarding GBV will clear up ambiguities, making the health care providers and the survivors more aware of the health professionals’ positions, and providing them with an opportunity for identification and disclosure.

*“They do not see it as their job. They see their interaction with their patient is to provide a certain service, and we do not see the extent to which GBV impacts. Some people do not take responsibility that it’s in their realm. So they think, oh, someone else will document that. That’s a social issue when really it is the responsibility of every care provider.”* (48 yr old Physician).

“*I think that is not their job, they are midwives and family doctors, and they just help the baby like delivering the baby.”* (35 yr old Survivor).

*“And, again, I think it is because it has been outside in the traditional realm of health care providers skills and knowledge base I think it is thought of more of like social work.”* (35 yr old Midwife).

#### Eliminate Time Constraints and Work Overload in the Healthcare System

From the participants’ point of view, violence and disclosure are very sensitive topics that require time and trust between the care provider and the survivor. They pointed out that care providers’ time constraints and workloads made women feel that the health care system was not ready to address GBV.

*“…when you feel devalued then if you were talking with medical professionals and they are overloaded you were not going to stand there that way.”* (35 yr old Survivor).

*“I think it is time restriction and exhaustion, burnout, and just feeling too overextended. Physicians, for example, do not spend as much time with clients as midwives do. So I imagine if you have a 10-min appointment, you’re not going to be solving all of these problems”.* (36 yr old Midwife).

### Main Theme #3: Providing Support for Survivors and Care Providers

Both care providers and survivors explained that they needed more support. Health care providers insisted on the importance of training, reducing their workload, and receiving compensation for service. Survivors also said that they needed more information. They mentioned that they could not afford to pay the fees for counselling and that this was one of the barriers to disclosure. Health care providers confirmed that the counselling fee prevented some survivors from seeking help.

#### Healthcare Provider Training

Most participants observed that healthcare providers were not sufficiently prepared to identify GBV and did not know the best supportive approach after disclosure. They believed that universities and health settings should make more effort to train care providers and provide them with instructions on dealing with GBV.

“My suggestion for the healthcare system is, keep learning, keep talking, and keep listening.

Treat the mental health of female patients as a priority, not as a hindrance or an afterthought.

So, I believe that would be the only solution I can see at this point.” (42 yr old Survivor).

“I have not had any formal training in addressing gender violence or partner violence. I have never been given any strategies of how to approach that conversation with women or men, or whatever their gender might be.” (29 yr old Nurse).

“I do not know how to start the conversation. How do I make them talk and explore their situation? I actually have been here for 20 years, and I have not formally attended one workshop that really shows me how to be prepared for it.”(47 yr old Nurse).

#### Support Healthcare Providers by Paying the Fee for the Service

In this study, physicians mentioned that performing assessment and providing support for survivors is time-consuming, and they should receive appropriate payment for this service. It was interesting that survivors were aware of this issue, and from their perspective, one of the reasons that physicians could not address GBV was not receiving adequate money for this service.

“The system needs to change its structure, we are going to create time within our system, and we are going to pay people to spend time talking to women about this issue. If you cannot pay your overhead cause you have to see a certain number of patients. You know. You are responsible for this” (48 yr old Physician).

“I think the biggest distance gap is how physicians are remunerated. Uh, the fee-for-service model does not facilitate people taking time to ask these questions or respond if the questions are positive”. (37 yr old Physician).

“So if my doctor takes more than 15 min to talk to me, he cannot bill for this extra time, which is why the clinics race through the people so fast. (41 yr old Survivor).

#### Providing Insurance Coverage for the Counselling Services

Providing support and counselling systems covered by insurance can encourage women to disclose their experience of violence. They need to be assured that disclosure would lead them to free support services. According to the interviews, the high cost of counselling and the lack of adequate insurance coverage led some women to refuse to disclose violence because they could not afford counselling fees.

“I have done a little bit of counselling, so I have talked to counsellors a little bit because counsellors are really expensive, and insurance does not cover counselling.” (41 yr old Survivor).

“The doctor did recommend some therapists to me, but I could not afford them.” (42 yr old Survivor).

## Discussion

The factors that may facilitate disclosure of GBV for pregnant and newly-parenting patients are complex and context-based. In this study, three main themes were identified that would each facilitate survivors of GBV in the perinatal period to disclose their experience: (1) Raising awareness about GBV; (2) creating shifts in the healthcare system’s approach to GBV; and (3) providing support for survivors and care providers.

“Raising Awareness”: For some of the survivors, these interviews were the first time they had spoken about the violence since being silenced. Survivors were unable to speak out about their experiences for many underlying reasons, including lack of knowledge and awareness, fear of disclosure and its consequences, not having access to resources and support, not having trust in the health care system, self-blaming and adverse reactions of people in their community and health care setting. Raising greater awareness of GBV might create contexts that support disclosure, where survivors would feel more comfortable speaking about their experiences. By raising awareness, the participants meant raising awareness in society, among pregnant women, and among perinatal healthcare providers. This finding is consistent with previous research, which has documented that raising awareness and fostering a better understanding of the consequences of GBV during pregnancy and the perinatal period could lead to the design of strategies aimed at preventing violence, increasing early detection, and more timely interventions (Taillieu et al., 2016). A Canadian qualitative study involving focus groups with health professionals reported that the public’s inherent trust in the medical profession facilitates care providers’ readiness to address GBV (Sprague et al., 2013).

To facilitate identification and disclosure of violence, the participants of their study suggested the need for raising awareness, possibly through posters in perinatal healthcare settings, increased education and training for perinatal healthcare providers, and providing resources for survivors (Sprague et al., 2013). In a study conducted in the UK, care providers’ attitudes towards survivors of GBV were generally positive. However, UK health care providers reported they only have a basic knowledge of GBV and that they needed more comprehensive training on this subject. They particularly mentioned that they need to have more information about available local services that could support survivors (Ramsay et al., 2012).

In addition to raising awareness, it is essential to pay attention to the underlying causes of GBV in society. The causes of GBV may vary based on the context and culture of each community. Therefore, designing programs and interventions should be developed based on the context (Perrin et al., 2019). Our study participants talked about care providers who believe in survivor’s stories and mentioned the importance of this tendency for disclosure. Validating women’s experiences, providing a safe atmosphere without judgment, and creating empathy could lead to more patient disclosure of GBV (Hegarty et al., 2020; Tarzia et al., 2020). There is growing evidence that women are willing to discuss experiences of GBV with healthcare providers (Tarzia et al., 2020; Usta et al., 2012). Also, women during their perinatal care may be more likely to disclose GBV, especially if the issue is raised by the care provider (O’Doherty et al., 2015). Therefore, healthcare providers should be trained to effectively identify, assess, and respond to GBV (Tarzia et al., 2020).

When our study participants were asked about underlying reasons for remaining silent and what would have facilitated disclosure, they explained that they were unsure about healthcare providers’ role regarding GBV. They were concerned that they would not be believed and validated by healthcare providers. In some situations, survivors who spoke out about their assault experiences said that they were ignored or devalued. These negative experiences seem to silence survivors. Our findings are consistent with the results of a study conducted in Sweden in which participants reported being concerned about the reactions of family, friends, and community to their disclosure and the effect of disclosure on their social interactions. The survivors who decided to speak up despite potentially negative outcomes referred to their need for emotional and practical support (Boethius & Åkerström, 2020). Perhaps when survivors are informed of the support they will receive from the healthcare system, they might be more inclined to disclose. As previously noted, the willingness of providers to hear about disclosure could be advertised through posters in perinatal health care settings, social media, and websites.

Study participants mentioned that Canadian/Western society is still patriarchal; some suggested that disclosure would become easier if men and women had equal rights and power. They suggested that gender inequality is a fundamental cause of GBV, a perspective supported by data that documents that gender inequalities increase the risk of violence by men against women and inhibit the ability of those affected to break the silence and seek help and protection (Health Promotion vs Health Protection, n.d.).

### Creating a Shift in the Healthcare System’s Approach Towards GBV

Study participants argued that the healthcare system’s policy towards GBV should be revised; they proposed that a shift in the model of care to relational practice should replace the current task-oriented approach. Evidence from other studies similarly mentions that fostering empathy and a positive relationship between survivor and healthcare providers would facilitate disclosure (Rose et al., 2011). Study participants also spoke about the importance of creating safety and confidentiality to support survivors to disclose experiences of abuse. Evidence shows that shame, fear of judgment, not being believed, and confidentiality concerns were among the main reasons for nondisclosure among survivors. Therefore, building trust, providing a safe atmosphere without judgment, and creating empathy could lead to more disclosure (Ahrens, 2006).

Healthcare providers should show their willingness to assist survivors safely while considering confidentially and respecting a woman’s right to choose whether or not to disclose a history of violence (Registered Nurses’ Association of Ontario, 2005).

Some of the participants from both groups in this study were not sure that addressing GBV was within the scope of medical practice. Evidence suggests that survivors and care providers need to know that dealing with GBV is a healthcare provider’s role (Usta et al., 2012). As noted, if l healthcare providers do not identify GBV as falling within their scope of practice, they may not become involved in looking for it and/or assessing patients for it, which would, in turn, limit women from disclosing their history of abuse (Usta & Taleb, 2014).

Rose et al. consider the assessment of GBV as one of the primary responsibilities of healthcare providers. They explain how disclosure will become easier for patients if care providers accept this responsibility and directly ask patients about any history of abuse. According to other studies, healthcare providers should be encouraged to be vigilant about the signs and symptoms of violence and engage in direct questioning while maintaining a supportive and secure environment (Doran & Hutchinson, 2017; Rose et al., 2011).

The healthcare providers in this study stated that they had not received specific training on GBV and felt they did not have enough knowledge to address it adequately. Therefore, they insisted on the crucial role of training and clarification of their practice scope. Training programs need to highlight the critical role of perinatal care providers regarding GBV assessment and intervention (Doran & Hutchinson, 2017). Our participants emphasized the role of empathy, asking questions and supportive interventions on the willingness of survivors to break their silence. Although screening may increase the rate of GBV identification during perinatal care, asking about the history of violence without further intervention and support does not protect women from more violence (Bair-Merritt et al., 2014). Therefore, effective and appropriate response and intervention towards women’s disclosure of GBV is the primary concern. According to a systematic review, although results of the interventions after disclosure in the included studies were heterogeneous, the majority of interventions demonstrated some benefits to survivors (Bair-Merritt et al., 2014).

Survivors preferred to access consistent perinatal care providers during their pregnancy and believed rotating care providers disrupted relationships during perinatal care. Having access to continuing care from the same personal physician is highly valued by family physicians and patients (College of Family Physicians of Canada, 2012). In line with our findings, other studies show that superficial relationships with care providers and fear of not being believed were among the main reasons for nondisclosure among survivors (Amin et al., 2017).

Finally, study participants suggested that looking at GBV through a multicultural lens without judgment would likely lead to more disclosure. Evidence confirms this finding by emphasizing the importance of considering the culture and sociopolitical dynamics of ethnic variation while approaching GBV (Kasturirangan et al., 2004).

Provide support for survivors and care providers: Our data suggest that both perinatal healthcare providers and survivors of GBV need additional supports from the healthcare system. Clinicians talked about needing training, less workload, and fees for providing care for GBV. Issues such as lack of time, insufficient skills, and feeling overwhelmed by the phenomenon’s emotional nature were mentioned by healthcare providers as barriers to adequately addressing GBV. Our findings have similarities with studies that refer to issues such as lack of time and insufficient skills as hurdles against disclosure. Our study identifies the facilitators to identification and disclosure as receiving information, having access to screening tools, skills training, and receiving support from the health care system that is equipped to manage GBV(Hegarty et al., 2020; Tarzia et al., 2020). Survivors also emphasized the need for care providers to spend more time listening to their stories; thus, decreasing the workload of health professionals and improving compensation for this type of work could create an extra time and give them the opportunity to address violence and provide conditions for disclosure (Hegarty et al., 2020; Tarzia et al., 2020).

Concerning work overload and lack of time, some existing evidence-based models of GBV intervention are compatible with busy health care settings. Nevertheless, none of the created interventions are primarily physician-delivered, and all are designed to be applied within the context of a multidisciplinary care team. (Bair-Merritt et al., 2014). Moreover, transforming primary care systems into Patient-Centered Medical Homes (PCMHs) could be beneficial in the identification of GBV and administration of appropriate interventions. In this regard, adding members responsible for responding to GBV in multidisciplinary teams could be helpful. Integrating interventions into primary care is another model. In this model, once GBV is identified, the survivor would be referred to an outside GBV advocate (Bair-Merritt et al., 2014).

Furthermore, based on perinatal health care providers experience, facilitators to identification and disclosure are receiving information, having access to screening tools, skills training, and receiving support from the health care system that is equipped to manage GBV(Hegarty et al., 2020; Tarzia et al., 2020). In line with our study’s findings, “Being supported by the health system” was one of the main themes that emerged from a meta-synthesis related to the readiness of care providers to address violence and abuse (Hegarty et al., 2020). Moreover, in their study, Beynon et al. confirmed that a supportive work environment is a valuable facilitator for the identification of GBV by care providers and would encourage survivors to disclose (Beynon et al., 2012).

Although visits to primary care providers and most hospital services are insured services in Canada, the study participants frequently mentioned the need for insurance coverage for counselling. It was clear from their experience that survivors would show more willingness to disclose if the counselling services were covered by insurance. Facilitating quick and easy access to counselling and providing the opportunity to discuss individual situations are important supportive strategies that would encourage survivors to talk about the history of GBV (Beynon et al., 2012).

### Recommendations

It seems that raising public awareness regarding GBV and the importance of disclosure and seeking help during the perinatal period might be enhanced through providing more information in public places, clinics, hospitals, and media. Also, informed and skillful health care providers who are aware of their critical role regarding GBV and are not overwhelmed by work overload would provide an atmosphere through which survivors feel comfortable disclosing. Knowledgeable and skillful perinatal care providers would have higher self-confidence for being involved in the identification of GBV. This goal would be attainable by paying more attention to GBV in schools and continuing education. Not only should healthcare providers be aware of their essential role concerning GBV, but also survivors and the community should know that this is in the realm of care providers’ practice. Moreover, campaigns that support the equal rights of men and women need to be strengthened in society. Women who break the silence and talk about their experiences should receive meaningful support. The healthcare system needs to reform from the inside, take practical steps regarding GBV, and be more equipped and prepared to support both women and care providers.

### Limitations

As this study took place over the course of the COVID-19 pandemic, this meant that in the middle of the project, our interviews shifted from in-person to telephone semi-structured interviews. Self-selection to the study is another limitation of the study, and the results are context-dependent to the Canadian healthcare system. The findings of this study are specific to the Canadian context. Also, the generalizability of the findings is limited because we excluded mothers who were current drug users or had major mental health problems like schizophrenia and major depression. We didn’t talk with health leaders in this study, and their perspectives regarding the facilitators of disclosure were not included in this study.

### Conclusion

This study suggests that the healthcare system needs to change its approach towards GBV. Care needs to be offered relationally and in safe conditions for survivors to feel comfortable to disclose and seek help. Clinical education and the healthcare system should invest in training and raising awareness regarding GBV, support restructuring the care encounter to build trust between survivors and care providers, and expand the relationship-based approach to healthcare. Perinatal healthcare providers need to be aware of their role and responsibility regarding GBV identification, know how to provide support, and be appropriately compensated for this work.
